# Liver Injury with Nintedanib: A Pharmacovigilance–Pharmacokinetic Appraisal

**DOI:** 10.3390/ph15050645

**Published:** 2022-05-23

**Authors:** Emanuel Raschi, Michele Fusaroli, Milo Gatti, Paolo Caraceni, Elisabetta Poluzzi, Fabrizio De Ponti

**Affiliations:** 1 Pharmacology Unit, Department of Medical and Surgical Sciences, Alma Mater Studiorum University of Bologna, 40126 Bologna, Italy; michele.fusaroli2@unibo.it (M.F.); elisabetta.poluzzi@unibo.it (E.P.); fabrizio.deponti@unibo.it (F.D.P.); 2 Department of Medical and Surgical Sciences, Alma Mater Studiorum University of Bologna, 40138 Bologna, Italy; milo.gatti2@unibo.it (M.G.); paolo.caraceni@unibo.it (P.C.); 3 SSD Clinical Pharmacology, Department for Integrated Infectious Risk Management, IRCCS Azienda Ospedaliero Universitaria di Bologna, 40138 Bologna, Italy; 4 IRCCS AOU di Bologna-Policlinico di S. Orsola, 40138 Bologna, Italy; 5 Center for Biomedical Applied Research, Alma Mater Studiorum University of Bologna, 40126 Bologna, Italy

**Keywords:** drug-induced liver injury, FAERS, spontaneous reporting system, pharmacovigilance, disproportionality, pharmacokinetics, prediction, nintedanib, pirfenidone

## Abstract

Drug-induced liver injury (DILI) with nintedanib has emerged as an adverse event of special interest in premarketing clinical trials. We characterized DILI with nintedanib in the real world and explored the underlying pharmacological basis. First, we assessed serious hepatic events reported to the Food and Drug Administration’s Adverse Event Reporting System by combining the disproportionality approach [reporting odds ratio (ROR) with 95% confidence interval (CI)] with individual case assessment. Demographic and clinical features were inspected (seriousness, onset, discontinuation, *dechallenge/rechallenge*, concomitant drugs) to implement an *ad hoc* causality assessment scoring system. Second, we appraised physiochemical and pharmacokinetic parameters possibly predictive of DILI occurrence. Significant disproportionality was found for nintedanib as compared to pirfenidone (N = 91; ROR = 4.77; 95% CI = 3.15–7.39). Asian population, low body weight (59 kg), and rapid DILI onset (13.5 days) emerged as clinical features. Hospitalization and discontinuation were found in a significant proportion of cases (32% and 36%, respectively). In 24% of the cases, at least two potentially hepatotoxic drugs (statins, proton pump inhibitors, antibiotics) were recorded. Causality was at least possible in 92.3% of the cases. High lipophilicity and predicted in silico inhibition of liver transporters emerged as potential pharmacokinetic features supporting the biological plausibility. Although causality cannot be demonstrated, clinicians should consider early monitoring and medication review on a case-by-case basis.

## 1. Introduction

Nintedanib is an orally administered antifibrotic tyrosine kinase inhibitor first approved in 2014 by the Food and Drug Administration (FDA) and the European Medicines Agency (EMA) for the treatment of idiopathic pulmonary fibrosis (IPF), and more recently (2019–2020) for systemic sclerosis-associated interstitial lung disease (SSc-ILD) and other chronic fibrosing interstitial lung diseases (ILDs) with a progressive phenotype.

The drug possesses various pharmacological properties, including inhibition of receptor tyrosine kinases implicated in the pathogenesis of ILDs, such as vascular endothelial growth factor receptors (VEGFR-1, -2, and -3), fibroblast growth factor receptors (FGFR-1, -2, and -3), and platelet-derived growth factor receptors (PDGFR-α and -β). Additionally, it inhibits the kinase activity of RET receptors, FLT3, and the Src family of tyrosine kinases, as well as transforming growth factor beta (TGF-β), thus suggesting potential effects on multiple signaling pathways and pleiotropic properties.

Nintedanib displays linear pharmacokinetics for a dose of 350 mg twice daily: bioavailability is 4.7% for a 100 mg dose, T_max_ is 2–4 h after oral administration with food, and the half-life is 9.5 h. The main metabolic pathway is esterase-mediated hydrolysis followed by glucuronidation, with CYP3A4 playing a minor role in biotransformation, and bile/fecal pathway as the main route of elimination accounting for 93% of the administered dose. No dose adjustments are required in patients with moderate renal impairment (creatinine clearance of 30–90 mL/min), while it is not recommended in case of moderate or severe hepatic impairment (the drug was not studied); a reduced 100 mg twice daily dosage is recommended in mild hepatic impairment (Child–Pugh A).

In multinational phase III trials, including open-label extensions, and real-world experience, the drug has demonstrated clinical benefit persisting over long-term treatment, with a manageable tolerability profile [1]. Nintedanib significantly reduced the annual rate of decline in forced vital capacity in patients with IPF and severe gas exchange impairment as well as in subjects with milder disease. Global pharmacovigilance data from Boehringer Ingelheim in IPF collected over 4 years (up to 15 October 2018) were consistent with the safety profile established in clinical trials; diarrhea had an incidence rate of 301.6 events/1000 person-years (PY), with most events being non-serious (97%) [2].

Apart from diarrhea, bleeding, and arterial thromboembolism, drug-induced liver injury (DILI), albeit uncommon, emerged as an adverse event (AE) of special interest [1]. Imbalances were noted in pivotal clinical trials: elevations in alanine aminotransferase, aspartate aminotransferase, or both to levels ≥ 3 times the upper limit of the normal range (ULN) were observed in 13.0% of the subjects with progressive fibrosing ILDs treated with nintedanib (vs. 1.8% in the placebo group), with only one patient in each group meeting criteria for Hy’s law [3]; the respective proportions were 4.9% vs. 0.7% in SSc-ILD [4], 4.9% vs. 0.5% in INPULSIS-1, and 5.2% vs. 0.9% in INPULSIS-2 trials [5], with no individuals meeting criteria for Hy’s law in IPF. In the Boehringer Ingelheim’s pharmacovigilance database, elevated liver enzyme or bilirubin levels occurred at a rate of 31.5 events/1000 PY, with a median onset of 60 days [2]. Liver enzyme and bilirubin elevations occurred within the first 3 months of treatment, were typically reversible with dose interruption or reduction, with certain patients at higher risk of elevations, including females, patients of Asian origin, or with low body surface area [6]. Therefore, liver function tests should be conducted before initiating treatment, during the first month of treatment, subsequent two months, and periodically thereafter [7,8].

Considering the expanding use of nintedanib, post-marketing surveillance is pivotal for early detection of clinically important hepatic events, as exemplified by a recent case report of suggestive acute hepatitis by nintedanib used for more than 4 months for suspected pulmonary fibrosis after coronavirus disease (COVID)-19 [9]. In this setting, pharmacology may be used to explore the potential underlying mechanisms of DILI, starting from estimating the extent of reporting in post-marketing surveillance, the so-called “bedside-to-bench” approach [10].

The aim of this study was to characterize the post-marketing reporting of DILI with nintedanib and investigate the underlying pharmacological basis. To this purpose, we implemented a two-step strategy based on (A) the analysis of spontaneous reports submitted to the FDA’s Adverse Event Reporting System (FAERS) database, focusing on hepatic reactions of clinical interest, namely rare but serious liver AEs with a recognized drug-attributable risk called designated medical events (DMEs), and (B) appraisal of physiochemical and pharmacokinetic features known to be potentially involved in DILI.

## 2. Results

### 2.1. Pharmacovigilance Analysis

Overall, 13,249 and 47,826 reports mentioning nintedanib and pirfenidone were found in the FAERS, with DILI cases accounting for 0.7% (N = 91, almost all as suspect) and 0.1% of the reports, respectively. A significant reporting odds ratio (ROR) was found for hepatic DMEs collectively [ROR = 4.77; 95% confidence interval (CI) = 3.15–7.39], including *hepatic failure* (N = 31; ROR = 5.18; 95% CI = 2.47–11.87) and *drug-induced liver injury* (58; 8.10; 4.30–16.59). Two cases of autoimmune hepatitis were also recorded. Ribociclib used as a positive control generated significant disproportionality for hepatic DMEs (110; 1.94; 1.59–2.35), including *hepatic failure* (52; 1.71; 1.28–2.25) and *drug-induced liver injury* (39; 2.98; 2.11–4.09).

Demographic and clinical data are presented in Table 1.

A peak was recorded in 2018 (N = 25), with ILDs being the most frequently reported reason of use (77.2%), followed by non-small-cell lung cancer (6.5%). The largest proportion of DILI cases was reported by clinicians (63.7% vs. 66.7% for pirfenidone), with a notable contribution from consumers (24.2% vs. 9.1%), and, remarkably, from Asia (44.0% vs. 6.1%), especially Japan (35.2%). Subjects aged > 65 years were especially represented (median age of 68), with male preponderance, and lower median weight (59 vs. 76 kg for pirfenidone). Hospitalization was recorded in 31.9% of the cases (vs. 18.2%), death—in 26.4% (vs. 42.4%). Discontinuation was reported in 36.3% of the DILI cases with nintedanib (vs. 30.3%), *dechallenge*—in 31.9% (vs. 18.2%), with two cases of positive *rechallenge*. A significantly higher proportion of concomitant drugs and comorbidities was also recorded.

The time-to-onset was 13.5 days (interquartile range (IQR), 5.75–44.75; plausible and calculated for 64 cases). Co-reported events were recorded in 75% of the cases, mainly diarrhea (N = 30). No co-reported drug reaction with eosinophilia and systemic symptoms were found. Concomitant hepatotoxic drugs were reported in a substantial proportion of the cases: 25% for group A (mainly simvastatin/atorvastatin, furosemide, sulfamethoxazole) and 35.9% for group B agents (mainly omeprazole/esomeprazole, rosuvastatin/fluvastatin, metformin). Paracetamol and immunotherapy (nivolumab, ipilimumab, pembrolizumab, atezolizumab) were recorded in seven and four cases, respectively. Ursodeoxycholic acid was reported in four cases, while herbals or food supplements with potential DILI liability, namely *Curcuma* spp. and *Monascus purpureus* (red yeast rice), were reported in two cases. In 24.2% of the cases, at least two hepatotoxic drugs were found (combining groups A and B and paracetamol). Causality assessment was highly probable for 9.9% of the DILI cases, probable/possible—for 82.4% of them (Supplementary Material).

### 2.2. Pharmacological Appraisal

The physiochemical and pharmacokinetic parameters retrieved for nintedanib and pirfenidone are summarized in Table 2.

Based on publicly available sources, nintedanib exhibited higher lipophilicity than pirfenidone (LogP, 3.7); however, cytochrome P450 (CYP) 3A4 isoenzyme accounted only for about 5% of hepatic metabolism. Of note, nintedanib is a substrate and a weak inhibitor of efflux transporter P glycoprotein (P-gp), whereas no in vitro effects are documented for organic anion-transporting polypeptide (OATP) 1B1, OATP1B3, OATP2B1, organic cation transporter (OCT) 2, multidrug resistance-associated protein 2 (MRP-2), or the efflux breast cancer resistance protein (BCRP).

Conversely, in silico tools predicted several interactions with the aforementioned transporters, including the bile salt export pump (BSEP). Moreover, they yielded a potentially higher risk of hepatotoxicity for nintedanib as compared to pirfenidone. The dose- and Cmax-based DILI scores were comparable (approximately 4–5), suggesting less DILI concern/weak evidence of hepatotoxicity, and similar to drugs with recognized DILI liability on the market (amiodarone, moxifloxacin).

## 3. Discussion

In the recent years, nintedanib has received accelerated assessment (EMA), priority review, orphan drug and breakthrough designations (FDA), with initial indication for IPF and extended marketing approvals in 2019 and 2020 for SSc-ILD and chronic fibrosing ILDs. This regulatory scenario makes post-marketing surveillance pivotal to timely characterize AEs of special interest such as liver injury, especially in the current COVID-19 era.

The occurrence of DILI with nintedanib was already identified in pivotal premarketing trials and initial spontaneous reporting data [1,2]: uncommon reversible elevation of transaminases mainly described in the first 3 months of treatment [7,8]. This study, for the first time, used a “hybrid” approach to estimate the post-marketing reporting of DILI with nintedanib and explored the underlying pharmacological basis, using pirfenidone as a comparator. This “bedside-to-bench” appraisal was recently proposed as an aid for both regulators and clinicians to promote safer prescribing and risk/benefit assessment [10].

The large-scale pharmacovigilance analysis of the FAERS detected a consistent disproportionality signal, indicating that the proportion of liver events was higher in subjects exposed to nintedanib as compared to pirfenidone, and corroborated the hypothesis that drug-specific susceptibility exists. Since the analysis of spontaneous reports cannot provide actual risk assessment, these findings ask for additional analytical studies, such as population-based investigations, to confirm the signal before any regulatory action can be envisioned. Of note, reporting of liver injury with nintedanib is unlikely to be affected by the so-called notoriety bias (i.e., increased reporting due to media attention) since no major regulatory warnings have been published by the FDA or the EMA; conversely, a direct healthcare communication with updated recommendations to prevent DILI was issued by the EMA in October 2020 for pirfenidone [11]. Liver injury is listed in the section “warnings and precautions” in the relevant prescribing information of both drugs.

Although DILI remains unpredictable, different clinical and pharmacological features emerged as the key findings of this study. First, the large proportion of DILI reports from the Asian population (an independent feature of ILDs), notably in subjects with low body weight, confirmed previous clinical evidence [1,6], thus strengthening the importance of carefully considering host-related risk factors in DILI susceptibility.

Second, we recorded a rapid median onset of liver events (2 weeks) and remarkable discontinuation and hospitalization, which underlines the need for stringent monitoring, especially in the aforementioned susceptible subjects. This latency is in line with a retrospective study on 32 Japanese subjects (62.5% not eligible for theoretical enrollment in INPULSIS trials); 11 subjects were discontinued due to increased transaminase elevation grade ≥ 2, with a median onset of 6 days [12]. Considering that liver function tests should be checked before initiating treatment, during the first month of treatment, and at regular intervals during the subsequent two months (and periodically thereafter as clinically indicated), we propose proactive stringent hepatic monitoring every 2 weeks for the first 6 weeks, in line with the protocol of pivotal trials, for timely dose reduction (transaminases of 3–5 ULN without signs/symptoms) or interruption (transaminases of >5 ULN or 3–5 ULN with signs/symptoms). Regular laboratory monitoring should also be considered thereafter since delayed DILI was described [13].

Third, concomitant drugs with hepatotoxic potential were recorded in almost half of the cases, suggesting that pharmacodynamic and pharmacokinetic drug interactions, as well as comorbidities, may have a contributing role in the occurrence of DILI in a large proportion of individuals. Clinical judgement is warranted on a case-by-case basis, and a careful medication review, early after nintedanib administration, can reduce the potential hepatotoxic burden of these co-medications and the relevant likelihood of DILI onset.

Fourth, physiochemical and pharmacokinetic features may explain, at least partially, the observed different reporting pattern between nintedanib and pirfenidone, including high lipophilicity and in silico predicted inhibitory activity on different hepatic transporters, including P-gp, BSEP, and BCRP. Moreover, two out of three in silico prediction tools yielded a risk of hepatotoxicity, including cholestasis, thus strengthening the need for further investigations to clarify the mechanistic basis of DILI, which remains unsatisfactory and largely unpredictable in clinical practice. We also support precise characterization of in vitro drug properties during premarketing development in order to fully address the spectrum of activity across various transporters possibly implicated in DILI onset.

When looking at the LiverTox database (https://www.ncbi.nlm.nih.gov/books/NBK547852/; accessed on 23 May 2022), a standard reference for clinicians, a likelihood score E (unproven but suspected cause of clinically apparent liver injury) is assigned to nintedanib (updated on 25 November 2017). Conversely, pirfenidone yields a likelihood score D (possible rare cause of clinically apparent liver injury) (updated on 24 June 2020). An update is warranted for these data. Moreover, this example exemplifies the paradox recently highlighted by Teschke and Danan between the promised quality and the actual data [14]. Apart from improving the definition and identification of DILI cases after the Roussel Uclaf causality assessment method (RUCAM) assessment, we also propose anchoring this website with (A) the aforementioned pharmacokinetic features, thus providing the predictive pharmacological basis; (B) the FAERS interactive dashboard, thus offering a global real-time pharmacovigilance picture as carried out in this study.

The potential use of nintedanib to counteract lung fibrosis in COVID-19 and relevant implications deserve a careful discussion. The incidence rate of pulmonary fibrosis can be estimated at 2–6% after moderate COVID-19 [15]. By virtue of their pleiotropic anti-inflammatory and antifibrotic actions and their favorable profile in terms of clinically relevant pharmacokinetic interactions when used in combination [16], nintedanib and pirfenidone have become the primary drug candidate in this setting, with several ongoing trials to assess their risk/benefit profile in COVID-19. However, different concerns might hamper the effective use of these drugs: their side effects partially overlap the symptoms of COVID-19 (e.g., diarrhea, fatigue, loss of appetite), thus hampering early diagnosis and worsening clinical manifestation. A key issue regards the timing of introduction of the antifibrotic treatment. In pivotal trials, the benefit of nintedanib started to be significantly different from placebo after the first 4–6 weeks of treatment, suggesting a quick onset of action in ameliorating profibrotic pathways. Moreover, the drug could also counteract the extensive endothelial damage in the acute phase of COVID-19 pneumonia. However, in more severe forms of COVID-19 in the intensive care setting, the addition of nintedanib to the standard of care can increase the risk of hepatotoxicity and synergize with anticoagulants to increase the bleeding risk [17]. A recent case report of acute hepatitis following nintedanib use for suspected COVID-related pulmonary fibrosis strengthened the crucial importance of differential diagnosis, detailed drug history, and regular liver monitoring [9].

We acknowledge the limitations of our study, related to both pharmacokinetic assessment and pharmacovigilance analyses, including the inability to calculate incidence and infer causality, potential existence of the remaining duplicates and reporting biases, lack of exposure data and comprehensive clinical features, including schedule/posology, laboratory (levels of transaminases) and instrumental data, as well as the underlying/pre-existing hepatic impairment, which do not allow fully applying the RUCAM. In this regard, the role of additional previous or concomitant drugs (e.g., P-gp inhibitors, undeclared herbals, history and extent of alcohol exposure) or other host-related risk factors cannot be ruled out with certainty. Notwithstanding the exploratory nature of these data, the worldwide catchment area of the FAERS supports the generalizability of the findings. Moreover, the individual case-by-case evaluation, inspired by customized causality assessment and supported by plausible physiochemical/pharmacokinetic properties, is a further call towards the development and validation of a predictive algorithm for DILI diagnosis.

## 4. Materials and Methods

### 4.1. Pharmacovigilance Analysis

We performed an observational retrospective pharmacovigilance study using the FAERS database (January 2004–September 2021), one of the largest publicly accessible repositories of AEs, comprising more than 15 million worldwide reports. The FAERS is especially suitable not only to investigate rare but serious AEs such as DILI, especially for newly approved medications or drugs with recently expanded therapeutic indications, but also to explore the underlying mechanistic basis (e.g., mitochondrial toxicity) [10,18].

To this purpose, publicly available quarterly data were downloaded (https://fis.fda.gov/extensions/FPD-QDE-FAERS/FPD-QDE-FAERS.html; accessed 1 March 2022) and preprocessed to remove duplicates (i.e., reports overlapping in the key prespecified fields, including active substance(s), AEs, event date, age, gender, reporter country, weight).

We first performed the so-called disproportionality approach; if the proportion of liver events is greater in the subjects exposed to nintedanib (cases), then in the subjects not exposed to this drug (non-cases), a disproportionality signal emerges [19]. Through this so-called case/non-case approach, the reporting odds ratio (ROR) with a relevant 95% confidence interval (95% CI) was calculated and deemed statistically significant by common thresholds (i.e., the lower limit of the 95% CI > 1 with at least three cases).

We adopted an active comparator design, i.e., by comparing nintedanib with pirfenidone, an anti-fibrosing agent approved in a comparable clinical setting and timeframe (IPF—in 2011 by the EMA and in 2014 by the FDA), and also previously associated with serious DILI occurrence [20,21]. Ribociclib served as the positive control: this kinase inhibitor employs a different mechanism of action as compared to nintedanib (CDK4/6 inhibitor) and was previously known to cause clinically relevant DILI due to pharmacokinetic features [22]. Considering the oncological use of ribociclib in metastatic breast cancer, disproportionality was calculated using reports by anticancer agents as a background for comparison to reduce the confounding by indication and provide a clinical perspective.

In order to prioritize and characterize hepatic AEs of clinical interest, we restricted the analysis to the so-called designated medical events (DMEs), i.e., serious AEs with a recognized by regulatory agencies drug-attributable risk: *acute hepatic failure, autoimmune hepatitis*, *DILI*, *hepatic failure*, *hepatic infarction*, *hepatic necrosis*, *hepatitis fulminant* [10,19].

Second, the selected cases were described in terms of demographic characteristics, namely age, sex, reporter country, seriousness (e.g., hospitalization), onset, discontinuation, *dechallenge/rechallenge* and then individually scrutinized for concomitant anti-hepatitis and hepatotoxic drugs; to this aim, the classification proposed by Björnsson et al. was used, focusing on agents in categories A and B (namely, drugs with ≥ 50 or 12–49 convincing reports in the published literature, respectively) [23]. These clinical features were combined to implement a customized causality assessment, adapted from the WHO–UMC system, a probabilistic algorithm. The cases were finally categorized as highly probable, probable, possible, unlikely (Supplementary Material).

### 4.2. Pharmacological Appraisal

By definition, DILI is idiosyncratic, i.e., the underlying mechanism is only partially understood, and different hypotheses have been formulated on the *primum movens*, ultimately resulting in mitochondrial damage [24,25]. However, various drug-related properties have recently been identified and proposed to increase DILI susceptibility by interacting with host factors in humans [24].

To explore the pharmacological basis of DILI with nintedanib (and pirfenidone as a comparator), we extracted the available physiochemical and pharmacokinetic features known to be potentially involved in DILI, namely threshold dose, lipophilicity, formation of reactive metabolites, oxidative stress, mitochondrial liability, hepatic metabolism, and inhibition of hepatic transporters [22]. Although there is debate on the actual predictive value, these properties are acknowledged by the EASL guidelines [25]. A recent review of 3312 DILI cases found that most implicated drugs (61.1%) assessed with the RUCAM are metabolized through cytochrome P450 isoforms [26].

Therefore, European public assessment reports provided by the EMA, DrugBank (https://go.drugbank.com/; accessed 1 March 2022), and in the published literature were used [27]. We also queried public online in silico prediction tools, namely ADVERPred (http://www.way2drug.com/adverpred/; accessed on 1 March 2022), Vienna LiverTox Workspace (https://livertox.univie.ac.at/; accessed on 1 March 2022), and VenomPred (http://www.mmvsl.it/wp/venompred/; accessed on 1 March 2022) to investigate hepatotoxic liability and the interaction profile with the liver transporters potentially involved in hepatic damage (accessed on 1 March 2022). Additionally, we applied the validated “rule-of-two” DILI risk model proposed by Chen et al. to predict and stratify the severity of DILI liability in humans [28].

## 5. Conclusions

This bedside-to-bench approach found over-reporting of serious hepatic reactions with nintedanib in post-marketing surveillance, which was supported, at least partially, by in silico predicted pharmacological properties.

Although causal association cannot be firmly inferred, we call clinicians to raise awareness about (a) the opportunity for proactive medication review of concomitant hepatotoxic drugs; (b) rare but early occurrence of DILI with nintedanib, especially in Asian poly-medicated subjects with low weight who may require clinical judgment on a case-by-case basis.

Considering the evolving use of nintedanib, possibly extending to COVID-19 lung fibrosis and other potential autoimmune ILDs, pharmacovigilance plays a key role in promoting targeted clinical surveillance and safer prescribing, especially for AEs of special interest such as DILI.

## Figures and Tables

**Table 1:** Demographic/Reporting data and clinical features.

	Nintedanib, DILI (N = 91)		Nintedanib, Other Events (N = 13,158)		Pirfenidone, DILI (N = 33)		Other DILI (N = 47,793)	
	N	%	N	%	N	%	N	%
***Demographic data***								
**Sex** *Female* *Male* *Missing*	37 50 4	42.53 57.47 -	4644 7696 818	37.63 62.37 -	12 20 1	37.50 62.50 -	22,713 19,332 5748	54.02 45.98 -
**Age distribution** *<18 years* *18-29 years* *30-49 years* *50-64 years* *65-74 years* *75-84 years* *≥85 years* *Missing*	0 0 7 23 27 18 3 13	0.00 0.00 8.97 29.49 34.62 23.08 3.85 –	4 16 260 1911 3608 3248 517 3594	0.04 0.17 2.72 19.98 37.72 33.96 5.40 –	0 0 1 7 8 7 1 9	0.00 0.00 4.17 29.17 33.33 29.17 4.17 –	2711 3364 9095 10,697 6260 3730 884 11,052	7.38 9.16 24.75 29.11 17.04 10.15 2.40 –
**Weight (kg)** *Median (IQR) [missing]*	59 (55–69) [37]		71 (59–84) [9,895]		76 (58–85) [24]		68 (56–82) [34,842]	
***Reporting data***								
**Type of reporter** *Consumer* *Healthcare practitioner* *Lawyer* *Other* *Pharmacist* *Physician* *Missing*	22 5 0 5 1 58 0	24.18 5.49 0.00 5.49 1.10 63.74 –	6269 641 1 757 589 4602 299	48.75 4.98 0.01 5.89 4.58 35.79 –	3 2 0 3 3 22 0	9.09 6.06 0.00 9.09 9.09 66.67 –	7609 3441 297 12,387 2375 18,674 3010	16.99 7.68 0.66 27.66 5.30 41.70 –
***Clinical features***								
**Seriousness criteria** *Death* *Life-threatening* *Disability* *Hospitalization* *Required intervention* *Congenital anomaly* *Other seriousness (unspecified)* *No seriousness specified*	24 5 2 29 0 0 31 0	26.37 5.49 2.20 31.87 0.00 0.00 34.07 0.00	2811 327 199 3864 1 3 2446 3507	21.36 2.49 1.51 29.37 0.01 0.02 18.59 26.65	14 3 0 6 1 0 9 0	42.42 9.09 0.00 18.18 3.03 0.00 27.27 0.00	17,164 4442 471 13,302 158 6 11,660 590	35.91 9.29 0.99 27.83 0.33 0.01 24.40 1.23
**Co-reported symptoms** *Median (IQR)*	3 (2–6)		3 (1–6)		3 (2–6)		4 (2–7)	
**Concomitant drugs** *Median (IQR)*	5 (2–11)		2 (1–6)		3 (1–6)		3 (1–7)	
**Comorbidities** *Median (IQR)*	4 (1–8)		2 (1–4)		1 (1–2)		2 (1–4)	
**DISCONTINUED ** **^†^**	33	36.30	NC		1	3.00	NC	

^†^ Based on the reported dates for the “end of treatment” or recorded *dechallenge/rechallenge*. DILI: drug-induced liver injury; IQR: interquartile range; NC: not calculated. Valid percentages were reported (missing data were not considered).

**Table 2:** Summary of the physiochemical and pharmacokinetic features potentially involved in DILI.

Feature	Pirfenidone	Criterium Fulfilled	Nintedanib	Criterium Fulfilled
***Physiochemical factors***				
**Molecular weight** *(>600 Da)*	185.2	No	539.6	No
**Scheduled daily dose** *(≥50–100 mg/day)*	801/1602/2403	Yes	300	Yes
**Lipophilicity** *(LogP ≥ 3)*	2.14	No	3.7	Yes
**Topological polar surface area** *(<75 Å2)*	20.31	Yes	94.22	No
**C_plasma_/BSEP** *(IC_50_) (≥0.1)*	No data	Unknown	No data	Unknown
***Oxidative stress and mitochondrial liability***				
**Reactive metabolites formation**	5-carboxy-pirfenidone Inactive	No	BIBF 1252/1053 Inactive	No
**“Rule-of-two”**	Negative	No	Positive	Yes
**Mitochondrial dysfunction**	No data	Unknown	No data	Unknown
***Metabolism and disposition***				
**Hepatic metabolism**	CYP1A2 +++ CYP2C9/2C19/2D6/2E1 +	Yes	CYP3A4 (<5%)	No
**BSEP inhibition**	No data (in vitro) Negative (0.00) (predicted in silico)	Unknown No	No data (in vitro) Positive (1.00) (predicted in vitro)	Unknown Yes
**MRP 2 transport**	No data (in vitro) Positive (0.60) (predicted in silico)	Unknown Yes	Negative (in vitro) Negative (0.20) (predicted in vitro)	No No
**MRP 3 inhibition**	No data (in vitro) Negative (0.00) (predicted in silico)	Unknown No	Negative (in vitro) Positive (1.00) (predicted in vitro)	No Yes
**MRP 4 inhibition**	No data (in vitro) Negative (0.00) (predicted in silico)	Unknown No	Negative (in vitro) Positive (1.00) (predicted in vitro)	No Yes
**P-glycoprotein inhibition**	Negative (in vitro) Negative (0.02) (predicted in silico)	No No	Weak (in vitro) Positive (0.97) (predicted in vitro)	Yes Yes
**BCRP inhibition**	No data (in vitro) Negative (0.07) (predicted in silico)	Unknown No	Weak (in vitro) Positive (0.61) (predicted in vitro)	Yes Yes
**OATP1B1 inhibition**	No data (in vitro) Negative (0.00) (predicted in silico)	Unknown No	Negative (in vitro) Positive (1.00)(predicted in vitro)	No Yes
**OATP1B3 inhibition**	No data (in vitro) Negative (0.00) (predicted in silico)	Unknown No	Negative (in vitro) Positive (1.00) (predicted in vitro)	No Yes
***DILI risk scores and prediction***				
**ADVERPred**	Negative	No	Negative	No
**Vienna LiverTox Workspace** * *Drug-induced liver injury* *Hyperbilirubinemia* *Cholestasis*	0.55 0.31 0.08	Yes No No	0.61 0.76 0.91	Yes Yes Yes
**VenomPred**	41% €	No	63% ±	Yes
**Dose-based DILI score** **	5.22	Less DILI concern/weak evidence	4.31	Less DILI concern/weak evidence
**Cmax-based DILI score** **	5.55	Less DILI concern/weak evidence	5.98	Less DILI concern/weak evidence

BSEP: bile salt export pump; BCRP: breast cancer resistance protein; MRP 2: multidrug resistance-associated protein 2; MRP 3: multidrug resistance-associated protein 3; MRP 4: multidrug resistance-associated protein 4; OATP1B1: organic anion-transporting polypeptide 1; OATP1B3: organic anion-transporting polypeptide 3; DILI: drug-induced liver injury; C_max_: peak concentration. * A score close to 1 indicates a high probability of causing DILI, hyperbilirubinemia, or cholestasis. A score close to 0 indicates a high probability of not causing DILI, hyperbilirubinemia, or cholestasis. ** Drugs receiving a score > 7, between 3 and 7, and <3 show, respectively, most DILI concern/solid evidence, less DILI concern/weak evidence, and no DILI concern/no evidence of hepatotoxicity; ± Predicted to be hepatotoxic, with low level of confidence; € Predicted to be non-hepatotoxic, with low level of confidence. “+++” means “major role”, whereas “+” means “minor role”.

## Data Availability

The datasets analyzed during the current study are publicly available (https://fis.fda.gov/extensions/FPD-QDE-FAERS/FPD-QDE-FAERS.html, accessed on 9 March 2022). Codes for the analyses are available upon reasonable request to the authors.

## Supplementary Materials

The following are available online at https://www.mdpi.com/article/10.3390/ph15050645/s1, Supplementary file S1: case-by-case assessment, Supplementary file S2: methods on the case-by-case assessment. Refs. [23,29,30,31,32,33] also list in Supplementary file S1.
