# Supplementary material for: Liver Injury with Nintedanib: A Pharmacovigilance–Pharmacokinetic Appraisal
Source: Pharmaceuticals (Basel). 2022 May 23;15(5):645. doi: 10.3390/ph15050645 (PMC9146184; doi:10.3390/ph15050645)
Supplement: Supplementary file 1 [file pharmaceuticals-15-00645-s001.zip › pharmaceuticals-1734792-Supplementary file S1.pdf]

SUPPLEMENTARY METHOD

CASE-BY-CASE EVALUATION: CAUSALITY ASSESSMENT ALGORITHM

Our causality assessment is an adaptation of the standardized WHO-UMC system, a probabilistic algorithm [1]. This algorithm and relevant criteria are conventionally used and recognized for causality assessment of spontaneous reports of adverse events, namely, to assess the drug-related component.

Assessment criteria are based on plausibility of time relationship to drug intake (time to onset), lack of concomitant diseases or drugs potentially explaining the event, plausibility of response to withdrawal (also known as *dechallenge*, namely clinical improvement after the offending agents is suspended), positive *rechallenge* (i.e., occurrence of a similar reaction after re-administration, usually unintentional) [2–5].

In the DILI scenario, the algorithm deserves careful adaptation, taking into account alternative causes such as non-toxic hepatitis and other hepatotoxic drugs. Therefore, we also considered the following additional domains/criteria: major and minor alternative causes (separately concomitant drugs and comorbidities), and the role of the drug in adverse event occurrence, as expressed by the reporter (i.e., if the drug was reported as suspect or concomitant). For the purpose of concomitant hepatotoxic drugs, we used the classification proposed by Björnsson et al. [6], who identified five categories of hepatotoxic drugs groups (A, B, C, D and E) based on the number of convincing reports in the published literature: category A, ≥50; category B, 12-49; category C, 4-11; category D, 1-3; and category E, none.

The obtained numerical score was converted to a categorical judgment of probability, namely *highly probable*, *probable*, *possible*, *unlikely*. Please note that, as compared to the original version, we decided to avoid the term "certain", considering that no firm causality can be inferred in pharmacovigilance through spontaneous reports.

References

1. The Uppsala Monitoring Center. The Use of the WHO-UMC System for Standardized Case Causality Assessment. Available online: https://www.who.int/medicines/areas/quality_safety/safety_efficacy/WHOcausality_assessment.pdf (accessed on 12 May 2022).
2. Sisi, M.; Fusaroli, M.; De Giglio, A.; Facchinetti, F.; Ardizzoni, A.; Raschi, E.; Gelsomino, F. Psychiatric Adverse Reactions to Anaplastic Lymphoma Kinase Inhibitors in Non-Small-Cell Lung Cancer: Analysis of Spontaneous Reports Submitted to the FDA Adverse Event Reporting System. *Target Oncol.* **2022**, *17*, 43–51.
3. Aiello, V.; Fusaroli, M.; Raschi, E.; Palazzini, M.; Hu, L.; Barbuto, S.; Poluzzi, E.; Capelli, I. Pulmonary Embolism in a Patient with ADPKD Treated with Tolvaptan: From the Clinical Experience to the Analysis of the Food and Drug Administration Adverse Event Reporting System Registry. *Kidney Int. Rep.* **2021**, *6*, 2472–2477.
4. Raschi, E.; Fusaroli, M.; Ardizzoni, A.; Poluzzi, E.; De Ponti, F. Thromboembolic Events with Cyclin-Dependent Kinase 4/6 Inhibitors in the FDA Adverse Event Reporting System. *Cancers* **2021**, *13*, 1758.
5. Raschi, E.; Fusaroli, M.; Ardizzoni, A.; Poluzzi, E.; De Ponti, F. Cyclin-dependent kinase 4/6 inhibitors and interstitial lung disease in the FDA adverse event reporting system: a pharmacovigilance assessment. *Breast Cancer Res. Treat.* **2021**, *186*, 219–227
6. Bjornsson, E.S.; Hoofnagle, J.H. Categorization of drugs implicated in causing liver injury: Critical assessment based on published case reports. *Hepatology* **2016**, *63*, 590–603.

SCORE ASSIGNMENT

Domain	Criterium	Notes and/or DILI customization
**Suspect (S)**	**0**: the drug is reported as concomitant by the reporter<br>**1**: the drug is suspected as a main cause for the event by the reporter	**1:** both primary suspect (PS) and secondary suspect (SS) were considered
**Dechallenge (D)**	**0**: no information<br>**1**: the event improves/regresses when the drug is withdrawn	
**Rechallenge (R)**	**0**: no information<br>**1**: the same/similar event reappears when reintroducing the drug	
**Concomitant Medications (I)**	**0**: no concomitant drugs recorded<br>**-2**: a concomitant drug known to cause the event is reported<br>**-1**: a concomitant drug possibly contributing to the event is reported	**-2**: explanatory hepatotoxic drugs (groups A and/or B), paracetamol overdose, alcohol<br>**-1**: facilitating hepatotoxic drugs (groups C and/or D), paracetamol therapeutic dose (<4 g/die)
**Comorbidities (P)**	**0**: no comorbidities recorded<br>**-2**: a comorbidity sufficient to cause the event is reported among the indications<br>**-1**: a comorbidity contributing to the event is reported among the indications	**-2**: hepatitis, cholangitis, metastases to liver, sepsis, shock. We considered also drugs as proxy of specific viral hepatitis (e.g., entecavir) but not the non-specific ones (e.g., valacyclovir)<br>**-1**: none considered
**Time to onset (latency)**	**0**: plausible time to onset or not calculated (missing data)<br>**-1**: time to onset not plausible (i.e., the event occurred before the drug was started)	Please note that, apart from excluding negative time to onset, we did not consider any strict interval to identify a plausible latency (there is no consensus on a pre-specified latency for DILI, since rapid, delayed cases are described, even after end of therapy)

FINAL CAUSALITY ASSESSMENT

**Highly Probable**	**2/3**: S_1D_1
**Probable**	**1**: S_1; $S_1D_1I_{-1}$; $S_1D_1R_1I_{-2}$; $S_1D_1T_{-1}$
**Possible**	**0/-1**: S_1I_{-2}; $S_1D_1I_{-2}$; S_1I_{-1}; S_1T_{-1}; T_{-1}; $S_1D_1I_{-2}T_{-1}$
**Unlikely**	**-2/-3/-4/-5**: $S_1I_{-2}P_{-2}$; $S_1I_{-1}P_{-2}$; $S_1I_{-2}T_{-1}$; $S_1D_1I_{-2}P_{-2}$
